# A 3D Genome Atlas of Genetic Variants and Their Pathological Effects in Cancer

**DOI:** 10.1002/advs.202408420

**Published:** 2025-03-25

**Authors:** Li Tang, Matthew C. Hill, Mingxing He, Junhao Chen, Zirui Wang, Patrick T. Ellinor, Min Li

**Affiliations:** ^1^ School of Computer Science and Engineering Central South University Changsha 410083 China; ^2^ Cardiovascular Research Center Massachusetts General Hospital Boston MA 02129 USA; ^3^ Cardiovascular Disease Initiative The Broad Institute of MIT and Harvard Cambridge MA 02142 USA

**Keywords:** 3D genome, cancer, Hi‐C, SNP, structure variants

## Abstract

The hierarchical organization of the eukaryotic genome is crucial for nuclear activities and cellular development. Genetic aberrations can disrupt this 3D genomic architecture, potentially driving oncogenesis. However, current research often lacks a comprehensive perspective, focusing on specific mutation types and singular 3D structural levels. Here, pathological changes from chromosomes to nucleotides are systematically cataloged, including 10 789 interchromosomal translocations (ICTs), 18 863 structural variants (SVs), and 162 769 single nucleotide polymorphisms (SNPs). The multilayered analysis reveals that fewer than 10% of ICTs disrupt territories via potent 3D interactions, and only a minimal fraction of SVs disrupt compartments or intersect topologically associated domain structures, yet these events significantly influence gene expression. Pathogenic SNPs typically show reduced interactions within the 3D genomic space. To investigate the effects of variants in the context of 3D organization, a two‐phase scoring algorithm, 3DFunc, is developed to evaluate the pathogenicity of variant–gene pairs in cancer. Using 3DFunc, IGHV3‐23′s critical role in chronic lymphocytic leukemia is identified and it is found that three pathological SNPs (rs6605578, rs7814783, rs2738144) interact with DEFA3. Additionally, 3DGAtlas is introduced, which provides a highly accessible 3D genome atlas and a valuable resource for exploring the pathological effects of genetic mutations in cancer.

## Graphical abstract

The hierarchical organization of the eukaryotic genome is vital for nuclear function, and disruptions from genetic mutations can alter this 3D architecture. Cataloging thousands of interchromosomal translocations, structural variants, and single nucleotide polymorphisms, their impact on 3D genome organization is revealed. The scoring algorithm, 3DFunc, and the comprehensive 3DGAtlas resource provide powerful tools and new insights into cancer‐related genetic alterations.

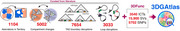

## 1 Introduction

To systematically elucidate the complex circuit of connections that exist between regulatory elements and genes, it is necessary to consider that interphase chromatin is folded in 3D in a cell‐type‐specific manner.^[^ ^1^, ^2^ ^]^ In recent years, chromosome conformation capture (3C) assays, in combination with next‐generation sequencing, have provided new insights into the global organization of the genome. Since the advent of these technologies, we have known that interphase genomic organization is multitiered. Each chromosome in the human genome occupies independent spatial territories, which play a role in the regulation of transcriptional activity and preferential positioning of loci within the nucleus.^[^ ^3^ ^]^ The analysis of Hi‐C data further refined the large scale of territories into two sets of mega‐base‐sized regions called “A” and “B” compartments.^[^ ^4^ ^]^ Compartment A is enriched in the regions of high gene density, active histone markers, early replication, and open chromatin.^[^ ^4^ ^]^ Compartment B has the opposite features, compared with A, and is associated with lamina‐associated domains, low transcriptional activity, and late replication, and hence is enriched in heterochromatin.^[^ ^4^ ^]^ The topologically associated domains (TADs) are mega‐base‐pair (Mb)‐sized genomic regions, and the TAD boundary regions contribute to the regulation of gene expression by limiting the interactions between *cis*‐regulatory elements and target genes. Finally, genomic loci can form specific long‐range looping interactions within or across TAD boundaries, through which the regulatory elements such as enhancers and insulators play a crucial role in controlling the gene expression profile of a cell in a context‐dependent manner.^[^ ^5^, ^6^ ^]^

At each layer in the genome organization hierarchy, the folding patterns exhibit a complex connection to the maintenance of genomic function, and mutations that effect any of these layers may lead to disease.^[^ ^7^, ^8^ ^]^ Some examples include the dissolution of chromosomal territories, as observed in both breast and prostate cancers.^[^ ^9^ ^]^ Altered genomic compartmentalization has also been reported in cancer cells. For example, by comparing normal breast cells (MCF‐10A) with their cancerous counterpart (MCF‐7 cells), it was found that a homogeneous switching of 12% of all chromosomal compartments would occur.^[^ ^10^ ^]^ Although the TAD structure has been found to be a general property of the interphase chromatin across different cell types, further studies have suggested that TADs are not simple stable interactions that are formed between two permanent genomic loci; rather, they are dynamic in nature.^[^ ^11^ ^]^ The deletion of the *Epha4* gene and CTCF associated boundary eliminates a TAD boundary, which causes the *Epha4* promoter to interact with the *Pax3* gene and drive misexpression of *Pax3*.^[^ ^12^ ^]^ Genomic duplication events can affect the expression of many genes, and spurious TAD formation can also lead to human disease.^[^ ^13^ ^]^ However, other studies have shown that the interruption of TAD boundaries has no obvious impact on gene expression.^[^ ^14^, ^15^ ^]^

A chromatin loop is formed when two distant genomic loci are physically closer than their intervening sequences. A classic example of long‐range gene regulation involves the *Shh* gene, the expression of which is regulated by an enhancer element ≈1 Mb away.^[^ ^16^ ^]^ Combining 3C techniques with genome‐wide association studies (GWAS) holds great potential for identifying new putative target genes/pathways for intergenic‐disease‐associated single nucleotide polymorphisms (SNPs). Recent studies showed that most disease‐associated SNPs reside within the regulatory elements and/or transcriptional factor binding sites in the noncoding regions of the genome and are likely to act through long‐range chromosomal interactions. Similar effects have also been shown in cases of prostate cancer, breast cancer,^[^ ^17^, ^18^ ^]^ and multiple other cancers.^[^ ^19^ ^]^

Current methods for assessing variant impact, such as CADD,^[^ ^20^ ^]^ fathmm‐MKL,^[^ ^21^ ^]^ and FunSeq2,^[^ ^22^ ^]^ provide valuable insights into variant pathogenicity by integrating diverse biological data. CADD evaluates both coding and noncoding variants through a holistic scoring system, fathmm‐MKL leverages multiple kernel learnings to predict variant effects using evolutionary and phenotypic data, and FunSeq2 focuses on prioritizing noncoding regulatory variants that may disrupt gene regulation.

In this study, we curate the pathological alterations from the chromosome level down to single nucleotides, including 10 789 interchromosomal translocations (ICTs), 18 863 structure variants (SVs), and 162 769 SNPs. We then analyze the 3D disruptions caused by these curated variants in four layers and observe that less than 10% of ICTs interrupted territories through strong 3D interactions, and very few SVs interrupted compartments or crossed TAD structures. However, these small‐scale events were found to impact gene expression significantly. Many SNPs residing within regulatory elements and/or in transcription factor binding motifs appeared to exert their effects by impacting loop strength. To assess the transcriptional impact of genetic variants within the framework of 3D genome organization, we introduce 3DFunc, a novel approach that goes beyond traditional tools by incorporating spatial genome architecture into its analysis. Unlike these methods, 3DFunc uniquely integrates gene expression data from 35 tissues with Hi‐C profiles from 33 tissues, allowing it to score the causality of genetic variants on transcription within a 3D genomic context. The application of 3DFunc to myelodysplastic syndrome (MDS) highlights its ability to detect causal variant–gene pairs under the context of 3D organization. Finally, we assemble a publicly available database, 3DGAtlas, to provide all the curated variants, the corresponding 3D layer disruptions, as well as the scoring results derived from 3DFunc.

## 2 Results

### 2.1 Disruptive ICTs with Strong 3D Interactions are Highly Pathogenetic

**Figure 1:**
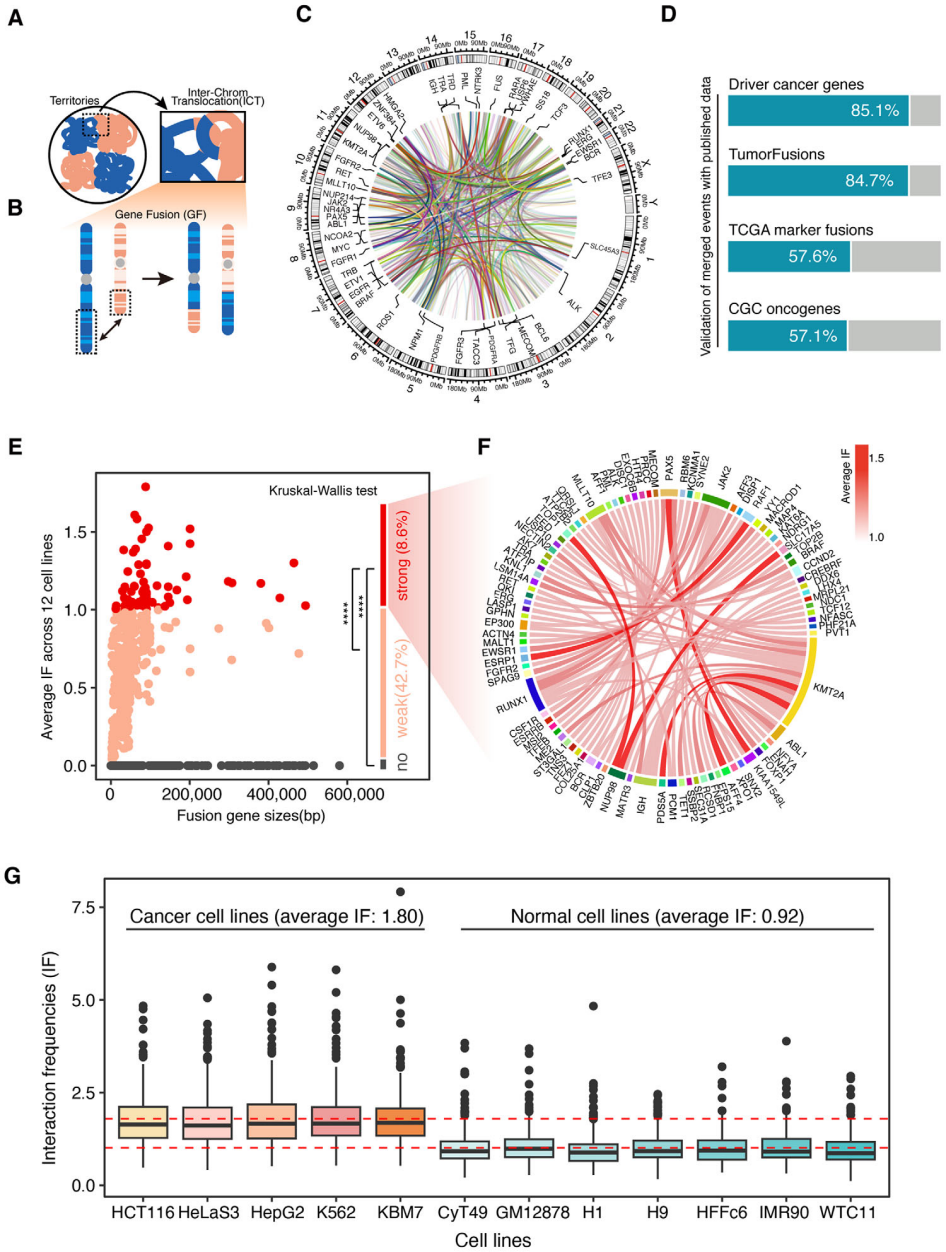
Curation of disruptive ICTs and correlation with 3D interactions. A,B) Diagram depicting that the ICTs interrupt territories, and ICTs lead to GFs. C) The overall distribution of high frequency fusion pairs (>10) across genome. D) Validation of merged fusion pairs. E) Average IF of merged fusion pairs across 12 cell lines. Difference between strong IF, weak IF, and no IF was calculated with Kruskal–Wallis test with Dunn test post‐hoc, **** *p*‐value < 0.0001. F) Circos plot of fusion pairs with strong 3D interactions. G) The IF values for individual cell lines, distinguishing between cancer and normal cells.

Genomic rearrangements are implicated in the pathogenesis of many types of cancer.^[^ ^23^ ^]^ Rearrangements occur when two or more double‐stranded DNA breaks are located proximally enough to fuse together (**Figure** **1**A), and recent studies have revealed that translocation can lead to gene fusions, dysregulated gene expression, and novel molecular functions^[^ ^24^ ^]^ (Figure 1B).

To investigate how interchromosomal translocations impact gene expression through spatial proximity, we curated 10 789 unique ICTs, which included 1104 unique gene fusions (GFs), and 1003 unique genes (Table S1, Supporting Information). There were 585 gene pairs appearing with high a frequency (>10) across the genome (Figure 1C). To validate the pathogenicity of merged events, we overlaid the involved genes with driver cancer genes from DriverDBV3,^[^ ^25^ ^]^ which showed an 85.1% overlap. The merged fusion events showed an 84.7% overlap with TumorFusions,^[^ ^26^ ^]^ a 57.6% overlap with fusions from TCGA markers, and a 57.1% overlap with oncogenes from the Cancer Gene Census^[^ ^27^ ^]^ (Figure 1D and Table S1 (Supporting Information)). Specifically, we identified the set of cancer‐related fusion gene pairs by overlapping fusion events from four published resources listed in Figure 1D. The results demonstrate that cancer‐related fusion gene pairs exhibit significantly higher interaction frequencies (IFs) compared to nonrelated gene pairs, reinforcing the link between these gene pairs and the distinct chromatin structure alterations observed in cancer cells (Figure S1, Supporting Information).

To correlate the pathogenic gene fusions with the spatial structure of genome territories, we calculated the flexible IF between each gene pair with high‐resolution Hi‐C data from 12 different cell lines. The results showed that 8.6% of the fusion pairs had strong 3D interactions with each other and 42.7% had weak 3D interactions (Figure 1E). Within the fusion pairs with strong 3D interactions, some of the pathogeneses have already been reported, such as *PAX5*, a transcription factor crucial for B‐cell commitment and maintenance, which typically fuses with KIAA1549L in childhood B‐cell precursor ALL.^[^ ^28^ ^]^ In pediatric acute leukemias, reciprocal chromosomal translocations frequently cause gene fusions involving the lysine (K)‐specific methyltransferase 2A gene (*KMT2A*); specific *KMT2A* fusion partners are associated with the disease phenotype (lymphoblastic vs myeloid). *MLLT10*, *PDS5A*, *AFF4*, and so on, are common fusion partners of *KMT2A* ^[^ ^29^ ^]^ (Figure 1F). Overall, only less than 10% of GFs interrupt chromosomal territories through strong 3D interactions; however, these GFs are highly correlated with pathogenesis. Furthermore, separate analyses across individual cell lines underscored cell‐type specificity: cancer cell lines consistently exhibited higher IF values than normal cell lines, reinforcing the potential oncogenic role of these 3D genome disruptions (Figure 1G).

### 2.2 Structural Variations Do Not Frequently Interrupt Compartment Switching but Do Cause Significant Changes in Gene Expression

**Figure 2:**
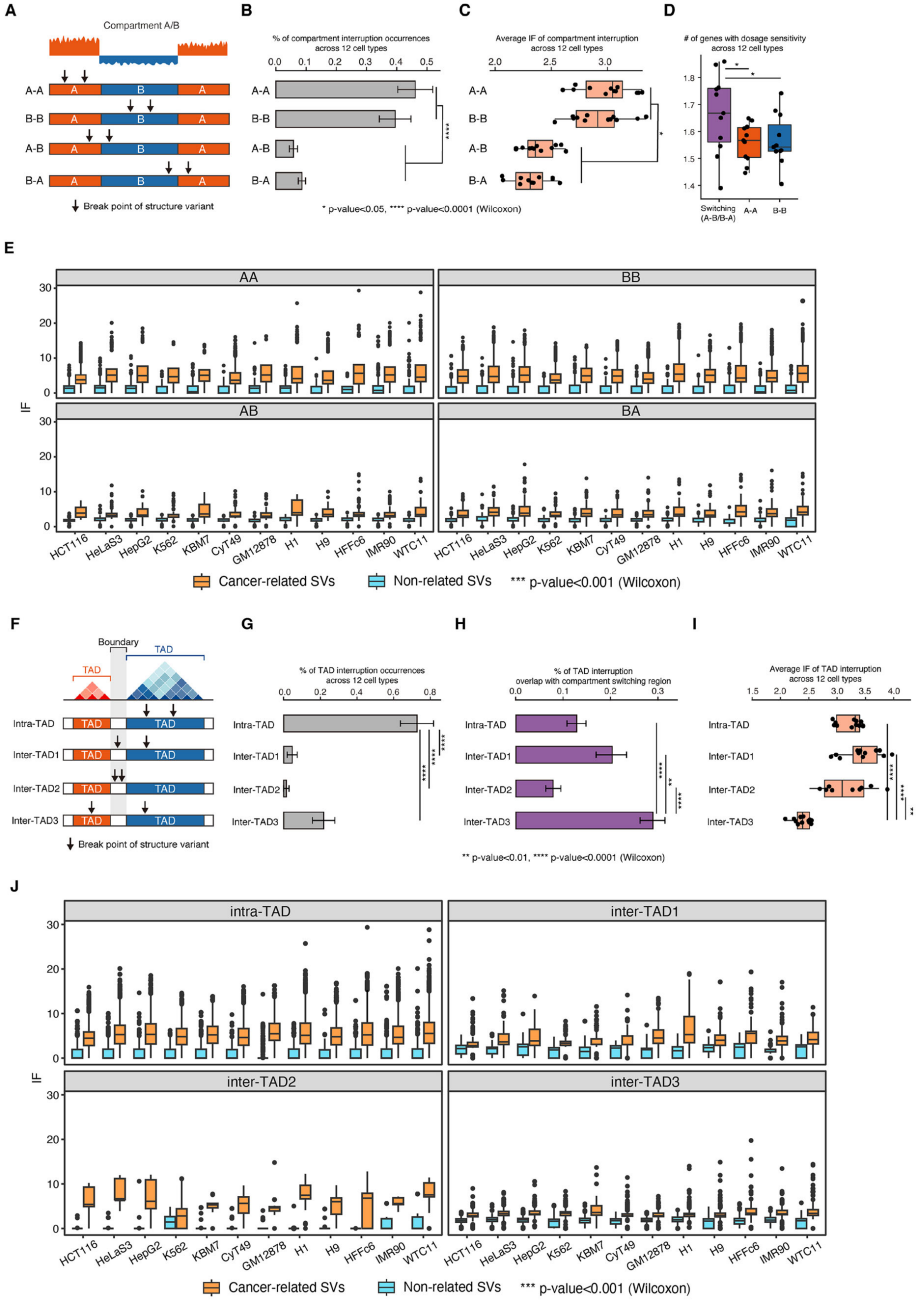
Interruptions of compartments and TADs caused by SVs. A) Four types of compartment interruptions: A–A (both breakpoints within A compartments), B–B (both breakpoints within B compartments), A–B (left breakpoint within A compartment and right breakpoint within B compartment), B–A (left breakpoint within B compartment and right breakpoint within A compartment). B) Percentage of compartment interruption occurrences across 12 cell types. C) Average IF of compartment interruption across 12 cell types. D) Number of genes with dosage sensitivity across 12 cell types for the interruptions in switching and stable compartment. E) Interaction frequency (IF) comparison for cancer‐related SVs and nonrelated SVs within stable and switching compartments. The differences were analyzed across 12 cell lines. F) Four types of SVs interrupt TADs: intra‐TAD (both breakpoints located within the same TAD), inter‐TAD1 (with one breakpoint locate in boundary and the other in TAD), inter‐TAD2 (both breakpoints located within the boundary), and inter‐TAD3 (two breakpoint locate in different TADs). G) Percentage of TAD interruption occurrences across 12 cell types. H) Percentage of TAD interruptions overlap with compartment switching regions. I) IF comparison for cancer‐related SVs and nonrelated SVs within TADs and across TAD boundaries. The differences were analyzed across 12 cell lines. Average IF of TAD interruption across 12 cell types. J) *p*‐value were calculated by Wilcoxon test, * *p*‐value < 0.05, ** *p*‐value < 0.01, *** *p*‐value < 0.001, **** *p*‐value < 0.0001.

We first examined 18 863 pathogenic SVs that included copy number gain, copy number loss, deletion, duplication, and insertion. We then filtered these SVs for length, selecting those with a size of 10 kb–10 Mb for the subsequent analysis (Figure S2, Supporting Information). Variants smaller than 10 kb are less likely to span entire regulatory units and therefore have a reduced potential to disrupt chromatin loops. By contrast, variants larger than 10 Mb are relatively rare and often encompass multiple TADs or even entire chromosomal arms, which complicates the identification of precise chromatin interactions. The filtered SVs were mapped to A/B compartments and four types of compartment interruptions including: A–A (both breakpoints within A compartments), B–B (both breakpoints within B compartments), A–B (left breakpoint within A compartment and right breakpoint within B compartment), B–A (left breakpoint within B compartment and right breakpoint within A compartment), in which A–A and B–B were defined as stable compartment interruptions, A–B and B–A were defined as switching compartment interruptions. We found that two breakpoints of SV occurred more frequently, especially in the type of A–A, within regions having no compartmental changes, while the SVs that did cause compartment switching, such as A–B or B–A, occurred in less than 20% of all SVs (**Figure** **2**A,B and Table S2 (Supporting Information)). Next, we calculated the flexible IF for all four possible types of compartmental disruption, and the stable compartment interruptions showed more 3D interactions than the switching types (Figure 2C and Table S2 (Supporting Information)). In addition, we observed that the SVs mapped to switching compartments involved more dosage sensitivity genes, consistent with a previous study that found that altered compartmental states can lead to transcriptional activity change^[^ ^30^ ^]^ (Figure 2D and Table S2 (Supporting Information)). And we analyzed RNA‐seq data from nine cell lines, focusing on the expression levels of genes located near SV breakpoints. We also examined whether these SVs were associated with compartment switching, the results show that switching compartments exhibit distinct expression patterns compared to those in stable compartments (Figure S3, Supporting Information). Our comparative analysis of cancer‐related and nonrelated SVs, sourced from ClinVar,^[^ ^31^ ^]^ COSMIC,^[^ ^27^ ^]^ dbVar,^[^ ^32^ ^]^ and TCGA,^[^ ^33^ ^]^ revealed that cancer‐related SVs had higher IFs in both stable and switching compartments (Figure 2E). Despite most SVs interrupting stable compartments with high 3D interactions, the small proportion causing compartment switching had greater transcriptional impacts with fewer 3D interactions.

Recent studies reported that structural variation can induce dramatic changes in TAD organization.^[^ ^12^, ^13^ ^]^ We mapped SVs to TAD boundaries and categorized the predicted interruptions into four categories, namely intra‐TAD (both breakpoints located within the same TAD), inter‐TAD1 (with one breakpoint located in the boundary and the other in TAD), inter‐TAD2 (both breakpoints located within the boundary), and inter‐TAD3 (two breakpoints located in different TADs) (Figure 2F). Among the four types of interruptions, intra‐TAD occupied the largest proportion, followed by inter‐TAD3 (Figure 2G and Table S2 (Supporting Information)). We then mapped the four types of TAD interruptions to the switching compartment regions. Inter‐TAD3 had the highest overlapping percentage with switching compartments, indicating that the inter‐TAD3 interruptions potentially correlated with greater transcriptional activity changes (Figure 2H). We then calculated the flexible IF for SVs in all four types of TAD interruption. Inter‐TAD3 showed the lowest 3D connection frequency (Figure 2I and Table S2 (Supporting Information)). Moreover, cancer‐related SVs showed higher IFs within and across TADs compared to nonrelated SVs (Figure 2J), confirming the heightened impact of cancer‐related SVs on 3D chromatin organization.

### 2.3 Analysis of Cancer‐Associated SNPs

**Figure 3:**
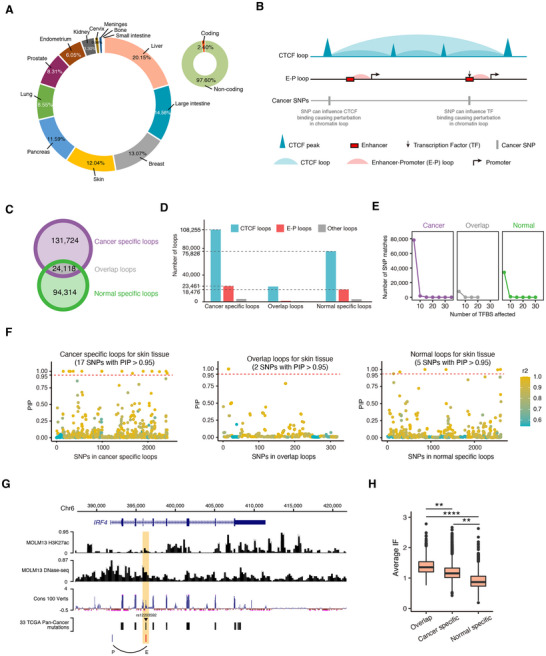
Analysis of cancer‐associated SNPs. A) Tissue distribution and coding region distribution of COSMIC cancer‐related SNPs. B) A schematic representation illustrating the influence of regulatory variants on TAD boundaries and intradomain interactions. The blue triangle represents the CTCF peak, the blue region signifies the area covered by the CTCF loop. The yellow triangle represents the H3K27ac peak, the red square symbolizes the enhancer, and the red area denotes the span of the E–P loop. Notably, cancer SNPs associated with transcription‐factor (TF)‐bound enhancers are accentuated, underscoring their pivotal role in modulating enhancer–promoter interactions. The diagram also emphasizes potential functional ramifications stemming from alterations that weaken TAD structural cohesion and chromatin interactions, potentially amplifying cancer susceptibility. C) Count of cancer‐specific loop (CSL), overlap loop (OL), and normal‐specific loop (NSL). D) Proportions of E–P loops, CTCF loops, and other loops within CSL, OL, and NSL. E) Quantity of enhancer's TFBS and the corresponding matched SNPs within CSL, OL, and NSL. F) PIP values in CSL, OL, and NSL of skin tissue. The red dashed lines represent cutoff of high PIP, the blue dashed lines represent the mean PIP. G) Representation of the chromatin loop between rs12203592 and the *IRF4* gene in CSL of skin tissue. H) Flexible IF computation for CSL, OL, and NSL. *p*‐value were calculated by Wilcoxon test, ** *p*‐value < 0.01, **** *p*‐value < 0.0001.

Then, we acquired 162 769 cancer‐related SNPs from COSMIC,^[^ ^27^ ^]^ spanning 13 distinct tissues. Among these, noncoding region SNPs accounted for 97.6% (**Figure** **3**A). Considering CTCF‐mediated chromatin looping leads to the formation of TADs, chromatin interaction analysis tools, such as ChIA‐PET^[^ ^34^ ^]^ and Hi‐C,^[^ ^35^ ^]^ have mapped these interactions and recognized TADs as large‐scale chromatin structures. CTCF or cohesin enrichment is typically observed at the TAD boundaries.^[^ ^36^ ^]^ These chromatin loops enhance intradomain interactions between regulatory elements, such as enhancers and gene promoters (which trigger gene expression). Concurrently, they restrict interdomain contacts to mitigate unspecific gene expression. In this model, regulatory variants at TAD boundaries or intradomain contacts could disrupt the protective neighborhoods formed by the looping, leading to aberrant enhancer–promoter interactions. Additionally, variants at active transcription‐factor (TF)‐bound enhancers can directly modulate these enhancer–promoter interactions. Alterations that compromise TAD structural integrity and chromatin associations are more probable to manifest functional consequences, potentially culminating in disease susceptibility (Figure 3B).

Here, we integrated available Hi‐C,^[^ ^35^ ^]^ ChIA‐PET/HiChIP,^[^ ^34^, ^37^, ^38^ ^]^ PCHi‐C,^[^ ^39^ ^]^ eQTL data,^[^ ^40^ ^]^ and CRISPR/Cas9‐verified loops^[^ ^41^ ^]^ across these tissues. By mapping curated SNPs to loops, a loop containing cancer‐related SNPs across all 13 tissues was categorized as a cancer‐specific loop (CSL). Conversely, a loop devoid of such SNPs in all tissues was deemed a normal specific loop (NSL). Otherwise, it was tagged as an overlap loop (potentially linked with both cancer relevance and nonrelevance) (Figure 3C). Moreover, we employed respective tissue CTCF ChIP‐seq peaks and DNase‐seq peaks for loop analysis, tallying the count of CTCF loops and enhancer–promoter (E–P) loops. The counting results revealed that across the three loop categories (CSL, overlap loop (OL), and NSL), CTCF loops predominated, followed by E–P loops. Specifically, CSL and NSL comprised 17.8% and 19.5% E–P loops, respectively, markedly surpassing OL (5.3%). We postulate this might be due to numerous contacts in the cancer and normal‐specific loops that remotely regulate cancer genes or sustain regular gene expression. By contrast, OLs seem minimally associated with gene regulation (Figure 3D). Subsequently, our investigation into the TF binding scenarios in E–P loops highlighted that the enhancer in CSL harbored up to 35 active TF binding sites (TFBS) and a maximum of 80 000 SNP matches, surpassing those in OL and NSL (Figure 3E).

The posterior inclusion probability (PIP) for the SNPs in skin tissue was computed using SuSiE^[^ ^42^ ^]^ to facilitate the determination of SNP causality. Notably, CSL SNPs displayed the highest average PIP (0.66), inclusive of 17 SNPs with high PIP (≥0.95) — a count surpassing those in OL and NSL (Figure 3F). As a case in point, we spotlight the intronic rs12203592 enhancer within the *IRF4* gene, which has ties with multiple phenotypic attributes, such as skin pigmentation and hair color. The chromatin loop in CSL involving this enhancer facilitates its interaction with the *IRF4* promoter, even if separated within the DNA sequence. It is paramount to note that the *IRF4* gene correlates with several hematological malignancies, including multiple myeloma, chronic lymphocytic leukemia, and non‐Hodgkin lymphoma. Variants within *IRF4*, exemplified by rs12203592, might influence these cancers' vulnerability or progression given their regulatory function^[^ ^43^ ^]^ (Figure 3G).

To investigate the interaction frequency of these SNPs with target genes in 3D structures, we calculated the flexible IF for each loop type. The findings indicated that OL exhibited the highest interaction frequency, followed by CSL, with NSL having the least frequency (Figure 3H), and IF comparison for CSL, OL, and NSL across 34 cell lines shows the same result (Figure S4, Supporting Information). In summary, SNPs with higher pathogenic potential tend to participate in more interactions within the 3D genomic space.

### 2.4 3DFunc: A Two‐Phase Scoring Algorithm for Detecting the Variants That Affect Gene Function through Long‐Range Genomic Interactions

To enlarge the testing datasets, we identified ICTs, SVs, and SNPs from the pan‐cancer analysis of whole genomes (PCAWG)^[^ ^44^ ^]^ (Figure S5, Supporting Information). First, we collected cell‐specific gene expression data from PCAWG and high‐resolution Hi‐C data from 4DN,^[^ ^45^ ^]^ then we ranked the identified variants with downstream expression changes and IF changes individually. The verified causal variants did not show a preferable top ranking with a single data type, indicating that the causality of variants was complex and could not be characterized with only a single data type (Figure S6, Supporting Information).

**Figure 4:**
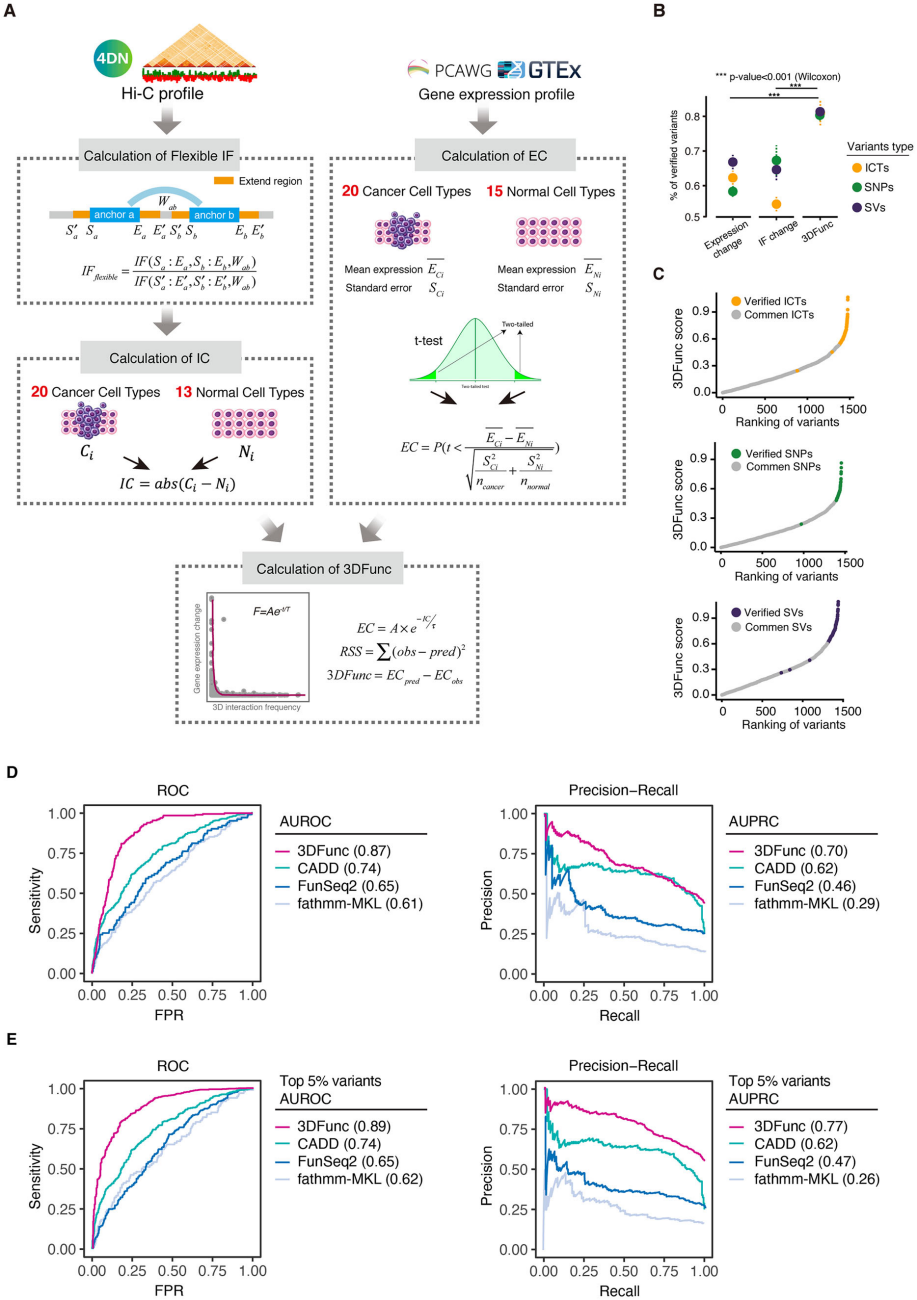
3DFunc identify the transcriptional effects of diverse sets of genetic variants. A) The schema of 3DFunc. B) The percentage of verified causal variants within the top 10% ranking parts, *p*‐value was calculated by Wilcoxon test, *** *p*‐value < 0.001. C) Rank variants with 3DFunc scores. D) Comparison of SNP scoring performance across tools using AUROC and AUPRC metrics. AUROC (left) and AUPRC (right) values were calculated for 3DFunc, CADD, fathmm‐MKL, and FunSeq2 to assess their accuracy in distinguishing pathological from nonpathological SNPs in the PCAWG dataset. E) Comparison of SNP scoring performance within the top 5% of ranked SNPs for 3DFunc, CADD, fathmm‐MKL, and FunSeq2, using the same AUROC and AUPRC metrics as in (D).

To address this challenge and quantify the effect of variants within the 3D genomic context, we developed a cell‐specific, two‐phase scoring algorithm, 3DFunc. First, 3DFunc employed Hi‐C matrices to compute the chromatin interaction frequency between predicted variants and their target genes. To address the low resolution of Hi‐C matrices, we expanded the anchor regions on both the left and right ends, and combined this with the anchor window size to calculate a flexible IF. Subsequently, 3DFunc computed the IF changes (ICs) between 13 normal samples and 20 cancer samples. For gene expression analysis, we used cell‐specific PCAWG data and applied a *t*‐test to evaluate the expression changes (ECs) of the mutated target genes between 20 cancer and 15 normal cell lines. By integrating both chromatin ICs and gene ECs, 3DFunc employed nonlinear least squares fitting to model the relationship between these variables for each cell line. Finally, the 3DFunc score was determined by calculating the difference between the expected and observed values for the fitted model, providing a comprehensive quantification of the variant effects in the 3D genome (**Figure** **4**A).

We used 3DFunc to score the predicted variants and calculate the percentage of verified causal variants within the top 10% of 3DFunc‐ranked variants. By cross‐referencing these top‐ranked variants with ClinVar^[^ ^31^ ^]^ annotations, we demonstrated that 3DFunc consistently prioritized verified causal variants, as SNPs labeled as “pathogenic” in ClinVar were highly enriched within the top 10% (Figure 4B,C). To further evaluate SNP scoring accuracy, we applied 3DFunc, CADD,^[^ ^20^ ^]^ fathmm‐MKL,^[^ ^21^ ^]^ and FunSeq2^[^ ^22^ ^]^ to SNPs from the PCAWG dataset. First, we mapped these SNPs to the ClinVar database; SNPs labeled as pathological in ClinVar were designated as positive samples, while others were considered negative samples. Using each tool's predicted scores, we calculated AUROC and AUPRC metrics to assess their performance in distinguishing pathological from nonpathological SNPs under both the original full dataset and the top 5% selection criteria. Results showed that 3DFunc achieved the highest AUROC (0.87) and AUPRC (0.70) among all tools when evaluated on the full dataset, demonstrating superior accuracy in prioritizing pathological SNPs (Figure 4D). To address concerns about the potential influence of false positives and dataset imbalances, we further restricted the analysis to the top 5% of variants predicted by each tool. Under this stricter criterion, 3DFunc maintained the highest performance, with an AUROC of 0.89 and an improved AUPRC of 0.77 (Figure 4E). By contrast, the performance of the other tools under the top 5% selection criteria showed minimal improvement or slight declines in AUPRC (CADD: 0.62–0.62, FunSeq2: 0.46–0.47, fathmm‐MKL: 0.29–0.26), indicating a lack of enrichment for true positive variants in their top‐ranked predictions. The results highlight the robustness of 3DFunc in prioritizing functional SNPs, even under conditions designed to reduce the potential influence of false positives.

### 2.5 Assessing the Efficacy of 3DFunc through Its Application to PCAWG Variants

**Figure 5:**
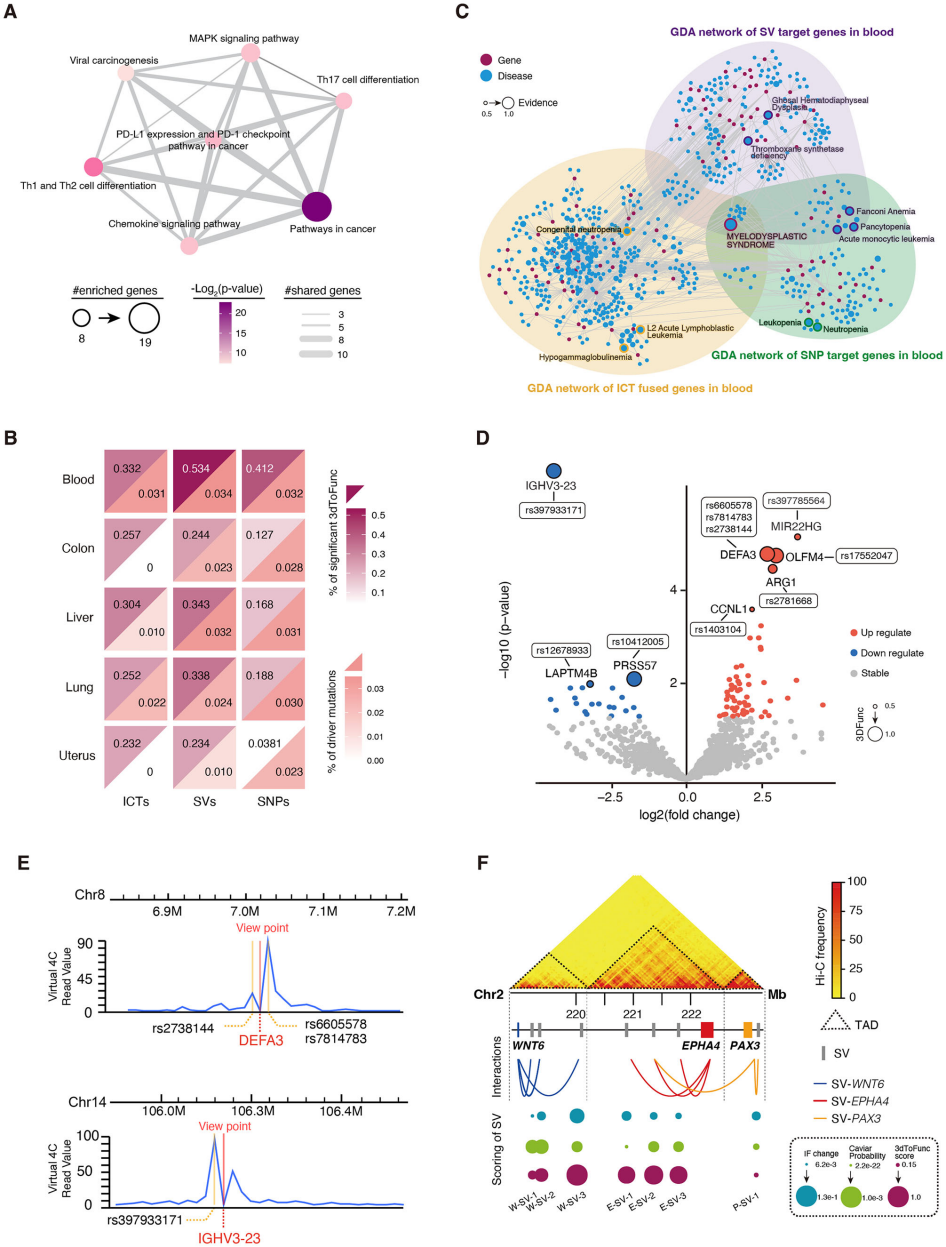
Evaluating 3DFunc's performance by applying it to PCAWG variants. A) KEGG pathway enrichment analysis of genes targeted by high‐scored SNPs. B) Percentage of significant 3DFunc scores, and the percentage of driver mutations in five tissues. The significance of 3DFunc scoring with chi‐square test in tissues of ICTs, SVs, and SNPs, and the overlapping percentage with the driver mutations from PCAWG. C) GDA network of ICTs, SVs, and SNPs in blood tissue. D) Differential expression analysis for TCGA MDS data with 3DFunc scoring, *p*‐value < 0.5 and the absolute value of log2(fold change) larger than or equal to 1 were used as the significant threshold. The purple points represent the genes, the blue points represent disease, and the size of points represent the evidence of gene–disease association. E) Virtual 4C analysis of chromatin interactions involving high‐scored SNPs near *IGHV3‐23* and *DEFA3*. F) Example of 3DFunc score in the locus of *WNT6*/*EPHA4/PAX3* gene. The heatmap indicated the Hi‐C profile and colored by interaction frequency. The triangle represented the regions of TAD structure. The gray squares represented SVs, and the curves represented SV–gene interactions. The IF change, caviar probability, and 3DFunc scores for each SV was marked with different colors. W‐SV‐1, W‐SV‐2, and W‐SV‐3 indicate the SVs near WNT6. E‐SV‐1, E‐SV‐2, and E‐SV‐3 indicate the SVs near EPHA4. P‐SV‐1 indicate the SV near *PAX3*.

To further investigate and validate the functionality of the predicted high‐scored SNPs (those with a 3DFunc score greater than 0.9 and *p*‐value less than 0.05), we conducted comprehensive analyses using publicly available datasets. First, we extracted variant–gene pairings from PCAWG for five prevalent tissues with both Hi‐C and expression data accessible. We then determined the significance of the 3DFunc scores using a chi‐square test. KEGG pathway enrichment analysis of the target genes of these SNPs revealed significant enrichment in cancer‐related pathways, such as “Pathways in Cancer,” “MAPK Signaling Pathway,” and immune‐related pathways including “Th1 and Th2 Cell Differentiation” and “PD‐L1 Expression and PD‐1 Checkpoint Pathway in Cancer” (**Figure** **5**A).

Next, we overlaid the driver mutations from the PCAWG dataset with the variants from common tissues in Hi‐C and RNA‐seq expression datasets. The variants in blood tissues showed the most significant 3DFunc scores (*p*‐value < 0.05) and the highest driver mutation rate (Figure 5B). To investigate the potential pathogenesis of these high‐scored SNPs, we further analyzed the Gene Disease Association (GDA) network for the target genes of these variants. We observed that MDS was most frequently associated with variants showing the highest evidence of disease risk (Figure 5C and Table S3 (Supporting Information)).

Additionally, we analyzed TCGA gene expression data and identified 85 differentially expressed genes between control and MDS samples. Notably, *IGHV3‐23* and *DEFA3* exhibited high 3DFunc scores. *IGHV3‐23*, a gene frequently mutated in chronic lymphocytic leukemia,^[^ ^46^ ^]^ and *DEFA3*, which is highly expressed in neutrophils, were found to interact with high‐scored SNPs, further supporting their functional relevance (Figure 5D and Table S4 (Supporting Information)).

To further evaluate the chromatin interactions associated with these SNPs, we performed virtual 4C analysis using *IGHV3‐23* and *DEFA3* as viewpoints. This analysis revealed strong interaction peaks near these genes, suggesting that the SNPs in these regions may influence chromatin looping and regulatory mechanisms (Figure 5E). Finally, we used eQTL data from the GTEx database to demonstrate significant associations between SNPs near *IGHV3‐23* and *DEFA3* and the expression of these genes across various tissues. For example, SNP rs2738144 near *DEFA3* and rs397933171 near *IGHV3‐23* exhibited strong eQTL signals, highlighting their potential role in modulating gene expression (Figure S7, Supporting Information).

In addition to SNPs, the PCAWG dataset also includes SVs, which we utilized to further verify the effectiveness of 3DFunc in scoring structural variants. We focused on high‐scored SVs and their corresponding interactions within the *WNT6*, *EPHA4*, and *PAX3* loci. Using Hi‐C data from H1 cells,^[^ ^35^ ^]^ we annotated the TAD structures at these loci. Among the *WNT6*‐related SVs, W‐SV‐3 (the third nearest SV to *WNT6*) exhibited the highest 3DFunc score, particularly near the TAD boundary. Duplication of this region misplaces the *WNT6* gene closer to an enhancer element, potentially causing its misexpression.^[^ ^12^ ^]^ For the SVs of *EPHA4*, three 3DFunc scores were larger than 0.5, indicating the causal effect of the upstream enhancer region of *EPHA4*. For comparison, we also showed the scoring of the Caviar probability, a measure of causal variants in associated regions,^[^ ^47^ ^]^ which did not display higher scores for the SV‐*WNT6* and SV‐*EPHA4* pairs (Figure 5F).

### 2.6 A 3D Genome Atlas of Genetic Variants and Their Pathological Effects

**Figure 6:**
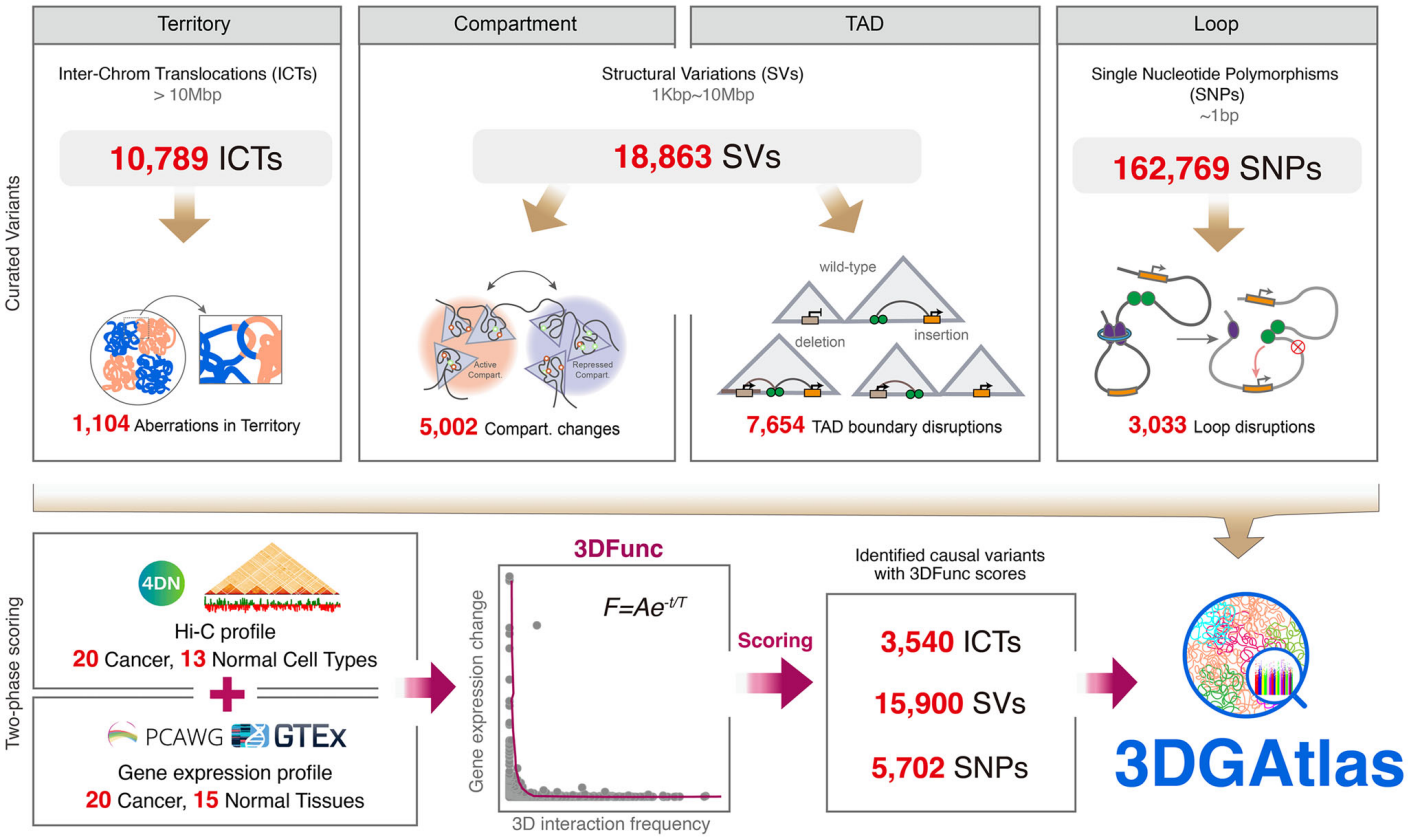
Design of 3DGAtlas. A) 10 789 ICTs, 18 863 SVs, and 162 769 SNPs were curated from literatures, in which 1104, 5002, 7654, and 3033 events were detected disrupting the corresponding 3D architecture layers. B) Hi‐C profile from 33 tissues and gene expression data from 35 tissues were combined with a nonlinear least square curve to generate two‐phase scoring algorithm 3DFunc. The identified variants were scored with 3DFunc. The database 3DGAtlas was constructed with the literature curated data and the 3DFunc predictions.

In the above work, thousands of pathological genetic mutations were curated, and we analyzed the topological genomic disruptions caused by these curated variants (**Figure** **6**A), in which 1104 ICTs interrupted territories, 5002 SVs correlated with compartment switching events, 7654 SVs disrupted TAD boundaries, and 3033 SNPs disrupted loops. To interpret how these variants would impact gene expression through 3D interactions in a quantitative manner, we generated a two‐phase scoring algorithm known as 3DFunc, which combines 33 Hi‐C datasets from the 4DN data portal,^[^ ^45^ ^]^ the gene expression data of 20 cancer tissues derived from the PCAWG,^[^ ^44^ ^]^ and 15 normal tissues taken from the GTEx portal.^[^ ^48^ ^]^ 3DFunc employs a nonlinear least‐square curve to measure the effect of genetic variants on long‐range gene regulation and genomic architecture. To demonstrate the effectiveness of 3DFunc, more ICTs, SVs, and SNPs were identified from the PCAWG datasets and scored with 3DFunc. Finally, we integrated all of the curated data and the calculated the 3DFunc scoring results to the 3DGAtlas database, in order to provide an atlas of genetic variants and their pathological effects for further research. This is available at https://www.csuligroup.com/3DGAtlas/home (Figure 6B).

## 3 Discussion

In recent years, 3C assays in combination with high‐throughput sequencing, such as Hi‐C, have provided new insights into the global organization of the genome. Since the advent of these techniques, we have known that interphase chromosomes are folded into four layers of hierarchical structures, including territory, compartment, TAD, and loop. The alterations in any of these layers can lead to disease or phenotype, thus, we curated the variants and mapped them to the 3D layers to investigate the pathological effects.

For territory, ICT could lead to gene fusions and dysregulated gene expression through spatial proximity. We observed that the fused gene pairs with more 3D interactions tended to corelate with pathogenesis. It is reasonable, as DNA FISH studies have revealed, that the frequency of translocations between two chromosomes is related to their spatial proximity in interphase nuclei.^[^ ^49^, ^50^ ^]^ Consistent with this finding, the 3D interaction counts from the Hi‐C profile between whole chromosomes were found to exhibit a strong correlation with the frequency of translocation, suggesting that spatial genomic proximity precedes translocation.^[^ ^51^ ^]^

For compartment, SV influences the expression of genes distant from the SV breakpoints and causes disease. In this study, most of the pathogenic SVs interrupted stable compartments, which also involved more 3D spatial connections. Although a small part of SVs interrupt switching compartments, these SVs impacted more gene expression through fewer 3D interactions. The observed frequencies of A–A and B–B interruptions provide key insights into the stability of chromatin architecture. A‐type compartments, associated with active chromatin, and B‐type compartments, linked to more repressive chromatin, exhibit different interaction patterns due to their roles in the genome. The predominance of stable A–A and B–B interactions may suggest that disruptions in these regions could significantly impact the regulatory landscape, given the compartmentalized nature of chromatin. Previous studies have highlighted the importance of compartmental interactions in maintaining gene regulation and expression patterns across cell types. The higher frequency of these stable interruptions may be indicative of the robust nature of chromatin domains that maintain their structure even in the presence of genetic aberrations.^[^ ^11^ ^]^ This has been linked to the self‐organizing principles of the genome, where chromatin tends to preserve its domain architecture due to factors such as loop extrusion, cohesin activity, and CTCF binding.^[^ ^52^ ^]^ The A–A and B–B therefore represent a protective mechanism, ensuring that essential regulatory regions maintain their activity despite structural variations.

For TAD, fewer SVs occurred inter‐TAD, and the type of inter‐TAD3 (two breakpoint locate in different TADs) correlated more with switching compartment and had less 3D interactions. Previous studies have demonstrated that compartments and TADs are highly dynamic in nature and changes occurred in accordance with lineage and cell‐type specificity.^[^ ^53^ ^]^ What is more, the altered compartmentalization and interruptions of TAD boundaries have been reported in disease and complex traits,^[^ ^10^, ^13^ ^]^ which explained why we observed that the SVs occurred in switching compartment and inter‐TAD structures impacted more dosage gene expression. In addition, fewer 3D interactions were detected near these regions, which was possibly caused by the dynamic characteristics of switching compartments.

For loop, we observed that the cancer specific SNP–gene loops have higher 3D interaction frequencies and with higher causality. Meanwhile, most of the SNPs locate within noncoding regions. This is consistent with the previous cancer studies that showed that most of the disease associated SNPs reside within the regulatory elements and/or are enriched in transcriptional factor binding motifs in the noncoding region of the genome and exerts effects through long‐range chromosomal interactions.^[^ ^17^, ^54^, ^55^ ^]^

In addressing potential biases in the selection of ICTs and gene fusions, we took several measures to ensure that the datasets used were as representative as possible of the broader cancer landscape. Specifically, we utilized well‐curated datasets such as PCAWG and 4DN to minimize selection biases. These datasets provide a comprehensive overview of somatic mutations, structural variants, and gene fusions across multiple cancer types, ensuring the inclusion of a wide range of genomic alterations observed in diverse tumor types and clinical contexts.^[^ ^33^, ^44^ ^]^ However, we acknowledge that no dataset is entirely free from biases, particularly those introduced by the overrepresentation of certain cancer types or the technical limitations inherent in detecting ICTs in lower‐resolution assays. To further mitigate these potential biases, we applied rigorous inclusion criteria, focusing only on high‐confidence gene fusions that have been recurrently reported in multiple independent studies and cancer types.^[^ ^56^ ^]^ We also cross‐referenced known driver mutations and well‐characterized oncogenic translocations, ensuring that our analysis captured clinically significant and functionally relevant events, rather than rare or incidental findings. Furthermore, our approach to ICT and gene fusion mapping involved stringent filtering based on the size and frequency of chromosomal rearrangements, allowing us to reduce the likelihood of artifacts arising from technical noise in sequencing data.

## 4 Experimental Section

### Curation of ICTs

The cancer‐related ICT events were collected from three predominant chromosome aberration database: Mitelman,^[^ ^57^ ^]^ COSMIC,^[^ ^27^ ^]^ and ChimerPub.^[^ ^58^ ^]^ For the data from Mitelman database (https://mitelmandatabase.isb‐cgc.org/), only the structural aberrations of interchromosome were retained, and the karyotype of which were converted to chromosome coordinates with CytoConverter.^[^ ^59^ ^]^ For the data from COSMIC (https://cancer.sanger.ac.uk/cosmic), the structural genomic rearrangements were downloaded, and the interchromosomal translocations were filtered out. For the data from ChimerPub (https://www.kobic.re.kr/chimerdb/chimerpub), the translocation data with known chromosome positions were retained. Then, all the ICTs from these three databases were merged and duplications were removed, 10 789 ICTs were retained in total. 1104 gene fusion events were extracted from the filtered unique ICTs. All the chromosome coordinates were extracted under the reference of hg38.

### Curation of SVs

The pathological SVs were collected from Clinvar^[^ ^31^ ^]^ (release of 2021‐08‐13) under the reference of hg38 (https://ftp.ncbi.nlm.nih.gov/pub/dbVar/sandbox/dbvarhub/hg38/), the SVs were filtered with the percentage of larger than 1%, the filtered SVs included copy number gain, copy number loss, deletion, duplication, and so on. For the subsequent analysis, 18 863 SVs with length of 10k to 10 M bp were retained.

### Curation of SNPs

The cancer‐related SNPs were collected from COSMIC.^[^ ^27^ ^]^ To narrow the scope to pathological SNPs, the fine mapping eQTL data were downloaded from the recomputed datasets of eQTL Catalogue^[^ ^60^ ^]^ (https://www.ebi.ac.uk/eqtl/Data_access/), the credible sets of the same tissue from different studies were merged and the duplicated SNP–gene pairs were removed.

### Disruption of 3D Layers

For the layer of territory, the filtered unique gene fusions from curated ICTs were regarded as the disruptions of territory, and the cytobands of two fused genes were regarded as the locations where aberrations occurred.

For the layer of compartment, high‐resolution Hi‐C datasets (>1 billion reads) of 12 cell types were first used from 4DN data portal^[^ ^45^ ^]^ to assign active (A) or inactive (B) compartments genome‐wide by FAN‐C^[^ ^61^ ^]^ with the resolution of 1 Mb, and the genome sequence of hg38 was applied to calculate the average GC content, as GC content was previously shown to correlate well with compartmentalization.^[^ ^61^ ^]^ Then, the curated SVs were overlayed with the compartments of 12 cell types individually, the criteria for mapping included calculating the distance between SV breakpoints and the nearest compartment transitions, the spanning locations of SVs were regarded as the disruptions of compartments. There were four different disruption types, A–A (both breakpoints within A compartments), B–B (both breakpoints within B compartments), A–B (left breakpoint within A compartment and right breakpoint within B compartment), B–A (left breakpoint within B compartment and right breakpoint within A compartment).

For the layer of TAD, high‐resolution Hi‐C datasets (>1 billion reads) of 12 cell types from 4DN data portal were used to call the insulation domains by FAN‐C with the window size of 1 Mb. The downloaded Hi‐C file was normalized with ICE,^[^ ^62^ ^]^ then the resolution of the Hi‐C data was carefully adjusted to ensure the TADs could be accurately identified despite the presence of large SVs. The curated SVs were overlayed with the TADs of 12 cell types individually, the criteria for mapping included calculating the distance between SV breakpoints and the nearest TAD boundaries, the spanning locations of SVs were regarded as the disruptions of TADs. Additionally, a size‐based filtering approach was implemented to exclude regions where SVs exceeded 50% of the local TAD size, ensuring that disrupted regions did not skew the overall results. There were four interruption types: intra‐TAD, inter‐TAD1, inter‐TAD2, and inter‐TAD3.

For the layer of loop, the curated SNPs were assigned to the target genes with Hi‐C, PCHi‐C, fine‐mapping eQTL, and CRISPR/Cas9‐verified loops. The Hi‐C loops were identified with HiCCUPS,^[^ ^62^ ^]^ and the PCHi‐C loops were detected by MMCT‐Loop.^[^ ^63^ ^]^ The fine‐mapping eQTL was downloaded from GTEx,^[^ ^48^ ^]^ and CRISPR/Cas9‐verified loops were from Gasperini et al.^[^ ^41^ ^]^ The chromosome regions near SNPs were extended to 1 kb on both sides, then the extended locus‐gene pairs were regarded as loops, and the SNPs were regarded as the interruption within loops.

### Quantification of Dosage‐Sensitive Genes

SVs were first categorized based on the chromatin compartments they disrupted (AA, AB/BA, or BB) by mapping SV breakpoints to compartment boundaries identified through Hi‐C data. For each category, dosage‐sensitive genes located within the disrupted regions were identified by cross‐referencing with ClinVar annotations. The frequency of dosage‐sensitive genes affected by SVs within each compartment type across the cell lines was then calculated to quantify transcriptional changes potentially induced by these structural alterations. To assess the impact of these SVs on transcriptional activity, compartment shifts (e.g., from active to inactive compartments) were examined that could alter gene expression levels. SVs disrupting AB/BA compartments, for instance, were expected to have different regulatory effects than those interrupting AA or BB compartments.

### Calculation of Flexible IF

The flexible IF used Hi‐C matrix to measure the interaction frequency between two specific genomic loci. To make the calculation fit different scales of genomic length, a flexible strategy was used to determine the window size of interaction frequency. Two candidate regions were defined as *C_a_* and *C_b_*, respectively. The start point of *C_a_* was *S_a_*, the end point of *C_a_* was *E_a_*, the start point of *C_b_* was *S_b_*, the end point of *C_b_* was *E_b_*. The Hi‐C files created by juicer^[^ ^62^ ^]^ contained several prebuilt matrices with different resolutions, such as 1000, 5000, 10 000, 50 000, 100 000, and 500 000 bp. For the candidate regions *C_a_* and *C_b_*, the nearest resolution *R_a_* and *R_b_*, respectively, could be determined. The window size for calculating interaction frequency was set to *W_ab_* = min{*R_a_*,*R_b_*}. The interaction frequency between *C_a_* and *C_b_* was calculated by Straw,^[^ ^62^ ^]^ which was represented by IF (*S_a_*: *E_a_*, *S_b_*: *E_b_*, *W_ab_*).

Since most of the Hi‐C files were low‐resolution, which made the IF sparse, here, the candidate regions were extended as background to calculate the flexible IF. The extended start point of *C_a_* was *S*′_*a*_ = *S_a_* − *R_a_* α, the extended end point of *C_a_* was *E*′_*a*_ = *E_a_* + *R_a_* α, the extended start point of *C_b_* was *S*′_*b*_ = *S_b_* − *R_b_* α, the extended end point of *C_b_* was *E*′_*b*_ = *E_b_* − *R_b_* α. The extension coefficient *α* was set to $\left\{ \begin{array}{c} R_{a}/R_{b}\left(if\;R_{a}\;>\;R_{b}\right)\\ R_{b}/R_{a}\left(if\;R_{b}\;>\;R_{a}\right) \end{array}\right.$. Then, the flexible IF was calculated by

(1) $$Flexible\;\;IF=\frac{IF\left(S_{a}:E_{a},S_{b}:E_{b},\;\;W_{ab}\right)}{IF\left({S^{\prime }}_{a}:{E^{\prime }}_{a},{S^{\prime }}_{b}:{E^{\prime }}_{b},W_{ab}\right)}$$

For the ICT events, the loci of two fused genes were extracted as candidate regions for calculation. For the SV events, all the SV–gene pairs within the same TAD structure were considered as the candidate regions. For the SNP–gene pairs, the chromosome regions near SNPs were extended to 1 kb on both sides, then the extended locus‐gene pairs were regarded as candidate regions.

### Validation of Merged ICTs

To validate the pathogeneses of merged ICTs, the driver cancer genes were collected from DriverDBV3^[^ ^25^ ^]^ (http://driverdb.tms.cmu.edu.tw/download), then the genes involving in the ICTs were overlayed with the driver cancer genes, and the overlapping percentage was calculated. For the gene fusions from the whitelist of TumorFusions^[^ ^26^ ^]^ (https://tumorfusions.org/), the fusions from TCGA marker papers, and the TCGA fusions dataset,^[^ ^33^ ^]^ the curated gene fusions were overlayed with them regardless the fusion directions, then the overlapping percentage was calculated.

### Genomic Annotation of SNPs

To investigate the 3D interaction characteristics of SNPs within different genomic regions, the curated cancer‐related SNPs were annotated to coding regions with the coordinates of known genes from UCSC^[^ ^64^ ^]^ (release of 2021‐11‐16) and RefGene^[^ ^65^ ^]^ (release of 2021‐03‐01), and the noncoding SNPs did not locate in a coding sequence or within 10 bp of a splice site annotated in the RefGene. And the SNPs were annotated with high resolution maps of DHSs data.^[^ ^66^ ^]^ Then, SNP2TFBS^[^ ^67^ ^]^ (https://ccg.epfl.ch/snp2tfbs/) was employed to investigate the TFBS associated with coding and noncoding SNPs, respectively. The enrichment of TF and the number of affected TFBS were calculated by SNP2TFBS.

### Process of PCAWG and GTEx Data

For PCAWG gene expression data, a total of 1359 samples in 20 cancer tissues were used, the reads were aligned with the alignment tools of TopHat2^[^ ^68^ ^]^ (release of v2.1.1) and STAR^[^ ^69^ ^]^ (release of v2.7.10a). Read counts to genes were calculated using Htseq‐count^[^ ^70^ ^]^ with GFT file from GENCODE human release v38. Then, counts were normalized using fragments per kilobase of exon per million mapped fragments (FPKM) normalization and upper quartile normalization. The final expression values were given as an average of the TopHat2 and STAR‐based alignments.

For GTEx data, the expressions of 15 healthy tissues in 3274 samples from GTEx (phs000424.v4.p1) were analyzed with the same pipeline as PCAWG gene expression data to calculate the FPKM value for genes. The calculated expression data of each sample were assigned a unique aliquot.

### Classification of CTCF Loops and E–P Loops

Given that E–P loops were generally fewer in number but were more closely associated with regulatory functions, identifying E–P loops was prioritized in the analysis. The LoopPredictor algorithm^[^ ^55^, ^71^ ^]^ was first applied to classify all detected loops and those categorized as E–P loops were selected. For the remaining loops, an overlap analysis was performed with CTCF ChIP‐seq peaks; loops with at least one anchor overlapping a CTCF ChIP‐seq peak were classified as CTCF loops. The remaining loops, with no overlap, were designated as other loops.

Finally, the number of CTCF loops, E–P loops, and other loops across CSLs, NSLs, and OLs was quantified to facilitate a detailed comparison of loop types within each category.

### The Two‐Phase Scoring Algorithm: 3DFunc

To measure the effect of variants under the 3D context quantitatively, a two‐phase scoring algorithm, 3DFunc, was proposed which combined cell‐specific gene expression data from PCAWG and GTEx, as well as the Hi‐C matrix from 4DN. For each of the 20 cancer cell lines and their corresponding cancer types, the gene expression changes and 3D interaction frequencies were calculated separately.

To measure the gene expression changes between cancer and normal cell lines, the putative target gene *i* of predicted variants was mapped to the PCAWG expression data for the specific cancer cell line, matched to its unique “aliquot,” the average matched gene expression of target gene *i* in *n*_cancer_ cancer cell lines was $\bar{E_{C_{i}}}$, the standard deviation was $S_{C_{i}}$, and the average matched gene expression of target gene *i* in *n*_normal_ normal cell lines was $\bar{E_{N_{i}}}$, the standard deviation was $S_{N_{i}}$. 20 cancer and 15 normal cell lines were used, with expression change EC calculated independently for each cancer cell line using a *t*‐test

(2) $$EC=P\left(t<\frac{\bar{E_{C_{i}}}-\bar{E_{N_{i}}}}{\sqrt{\frac{S_{C_{i}}^{2}}{n_{cancer}}+\frac{S_{N_{i}}^{2}}{n_{normal}}}}\right)$$

To characterize the 3D interaction frequency between predicted variant and target gene, the flexible IF for each variant–gene in the corresponding cancer cell line was calculated. For putative target gene *i*, the flexible IF in a specific cancer cell line was C_*i*_, and the flexible IF in coresponding normal cell lines was N_*i*_, the flexible IF change was defined as IC = abs(C_*i*_  −  N_*i*_).

Next, nonlinear least square curve^[^ ^72^ ^]^ fitting procedure was employed separately for each cancer cell line to model the relationship between EC and IC. It was observed that the data likely followed an exponential decay pattern, leading to the fitting function

(3) $$EC=Ae^{-IC/\tau }$$

where *A* and τ were determined by the residual sum of squares (RSS)

(4) $$RSS=\sum {\left(obs-pred\right)}^{2}$$

The goal of nonlinear least squares fitting algorithm was to find function parameters that minimized the RSS. The curve‐fitting process was performed for each cancer cell line using a custom R script. The resulting model parameters were stored in an R object named “model.” The 3DFunc score was then calculated as the difference between the expected EC and the observed EC, tailored to each specific cell line.

### Scoring of Caviar Probability

To compare the scoring results of 3DFunc with other causal variants detecting methods, the Caviar Probability of the predicted variants was calculated with CAVIAR,^[^ ^47^ ^]^ which took GWAS and eQTL linkage disequilibrium files as input to detect the causal variants, the Colocalization posterior probability for each variant was used as the final Caviar Probability.

### Construction of GDA Network

The variant–gene pairs with the threshold of 3DFunc > 0.5 and *p*‐value < 0.05 were first filtered, then 100, 76, 116, 166, and 163 target genes were extracted from different blood, colon, liver, lung, and uterus individually. The GDA network was built with DisGeNET cytoscape app^[^ ^73^ ^]^ with the curated source, all the annotation types, and all the disease class. The score and EI threshold were set from 0.5 to 1.0. The output associations included 831, 603, 971, 1091, and 925 diseases for each tissue. The GDA network was grouped by the type of variants, and the nodes were resized by the detected evidence. Finally, the diseases related to the accordingly tissues were selected and marked on the GDA network.

### Analysis of TCGA MDS Data

For the TCGA MDS data, two cancer samples and two normal samples in BEATAML1.0‐COHORT were used, the gene counts were normalized by FPKM, which were taken as the input of DESeq2^[^ ^74^ ^]^ and the default parameters were used to detect differentially expressed genes. Then *p*‐value < 0.5 and the absolute value of log2(fold change) larger than or equal to 1 were used as the significant threshold to filter the genes, which showed 85 genes significantly differential expressed.

### Statistical Analysis

All statistical analyses were performed using R (version 4.3.1). Differences between strong IF, weak IF, and no IF groups were assessed using the Kruskal–Wallis test, with post‐hoc pairwise comparisons conducted using the Dunn test. The percentage of verified causal variants within the top 10% ranking was compared using the Wilcoxon test. Flexible IF computation was applied to CSL, OL, and NSL, with statistical significance assessed using the Wilcoxon test. ** *p*‐value < 0.01, *** *p*‐value < 0.001, **** *p*‐value < 0.0001.

### Data and Code Availability

The GEO datasets used in this study were available under the accession numbers: GSE116862,^[^ ^75^ ^]^ GSE51312,^[^ ^76^ ^]^ GSE66733,^[^ ^10^ ^]^ GSE71862,^[^ ^10^ ^]^ and GSE179128.^[^ ^77^ ^]^ The ArrayExpress database was under accession number of E‐MTAB‐2323, and AMP‐CMD accession number of TSTSR043623.^[^ ^78^ ^]^

All the curated variant–gene pairs along with the 3D interruptions, as well as the 3DFunc scoring results were available at the 3DGAtlas database: https://www.csuligroup.com/3DGAtlas/home.

The source code of 3DFunc was available at GitHub repository: https://github.com/CSUBioGroup/3DFunc. The data that support the findings of this study were openly available in figshare https://doi.org/10.6084/m9.figshare.26318707, reference number.^[^ ^79^ ^]^

## Conflict of Interest

P.T.E. received sponsored research support from the Bayer AG and IBM and had served on advisory boards or consulted for Bayer AG, MyoKardia, and Novartis. The remaining authors declared that they have no competing interests.

## Author Contributions

L.T. and M.L. conceived of the presented idea. L.T., M.H., and J.C. collected the data and designed the model, L.T. wrote the source code of 3DFunc. M.H. and J.C. developed the framework of database. M.C.H. helped improve the bioinformatics analysis. M.C.H., P.T.E., and M.L. aided in interpreting the results and provided input on the data presentation. All authors provided critical feedback and helped shape the research, analysis, and paper.

## Supporting information



Supporting Information

Supplemental Table 1

Supplemental Table 2

Supplemental Table 3

Supplemental Table 4

## Data Availability

The data that support the findings of this study are openly available in figshare at https://doi.org/10.6084/m9.figshare.26318707, reference number 79.
