# Supplementary material for: A 3D Genome Atlas of Genetic Variants and Their Pathological Effects in Cancer
Source: Adv Sci (Weinh). 2025 Mar 25;12(19):2408420. doi: 10.1002/advs.202408420 (PMC12097094; doi:10.1002/advs.202408420)
Supplement: Supplementary file 1 — Supporting Information [file ADVS-12-2408420-s004.docx]

**Supplemental Materials**

**A 3D genome atlas of genetic variants and their pathological effects in cancers**

Li Tang ^1^, Matthew C. Hill ^2,3^, Mingxing He ^1^, Junhao Chen ^1^, Zirui Wang ^1^, Patrick T. Ellinor ^2,3^, Min Li ^1*^

^1^School of Computer Science and Engineering, Central South University, Changsha 410083, China

^2^Cardiovascular Research Center, Massachusetts General Hospital, Boston, MA 02129

^3^Cardiovascular Disease Initiative, The Broad Institute of MIT and Harvard,

Cambridge, MA, USA 02142

***Corresponding Author:**

Min Li, e-mail: [limin@mail.csu.edu.cn](mailto:limin@mail.csu.edu.cn)


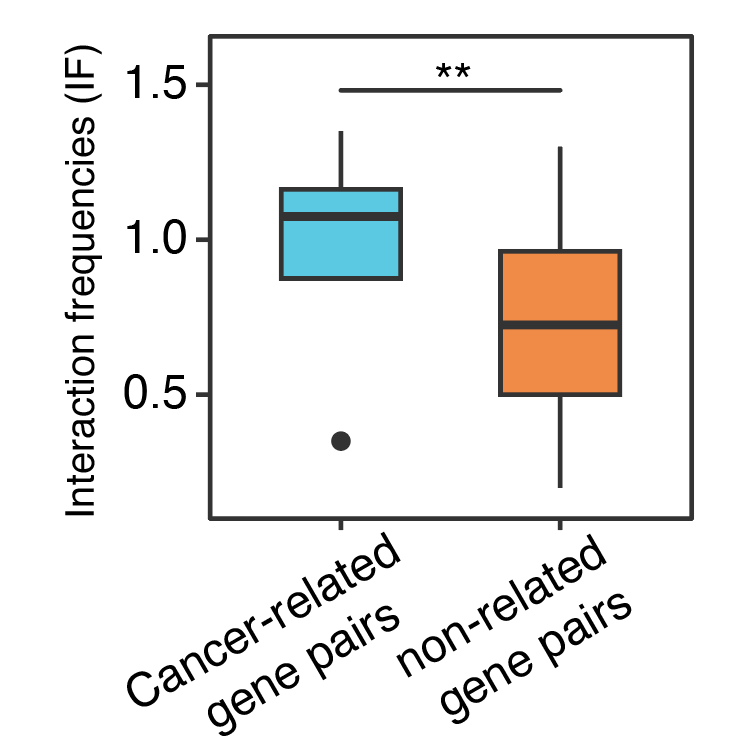


**Figure S1.** The interaction frequency (IF) comparison between cancer-related fusion gene pairs and non-related gene pairs. Difference was calculated with Kruskal-Wallis test with Dunn test post-hoc, ** p-value < 0.01.


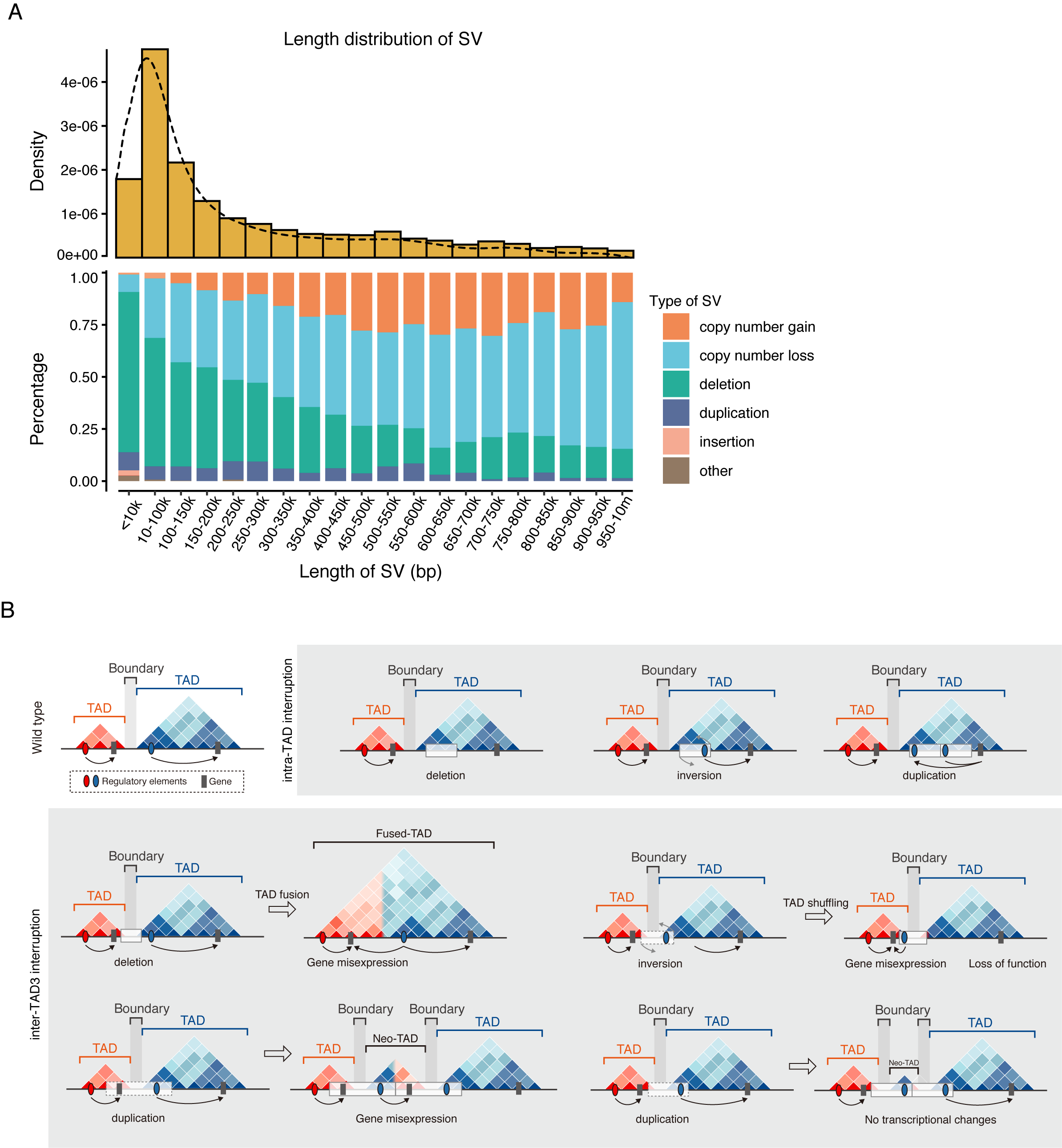


**Figure S2.** The length distribution of SVs (upper), and the percentage of different types of SVs (bottom). The type of insertion and other SVs only occupied small percentage, here we the SVs with length from 10k bp to 10m bp were used for the subsequent analysis.


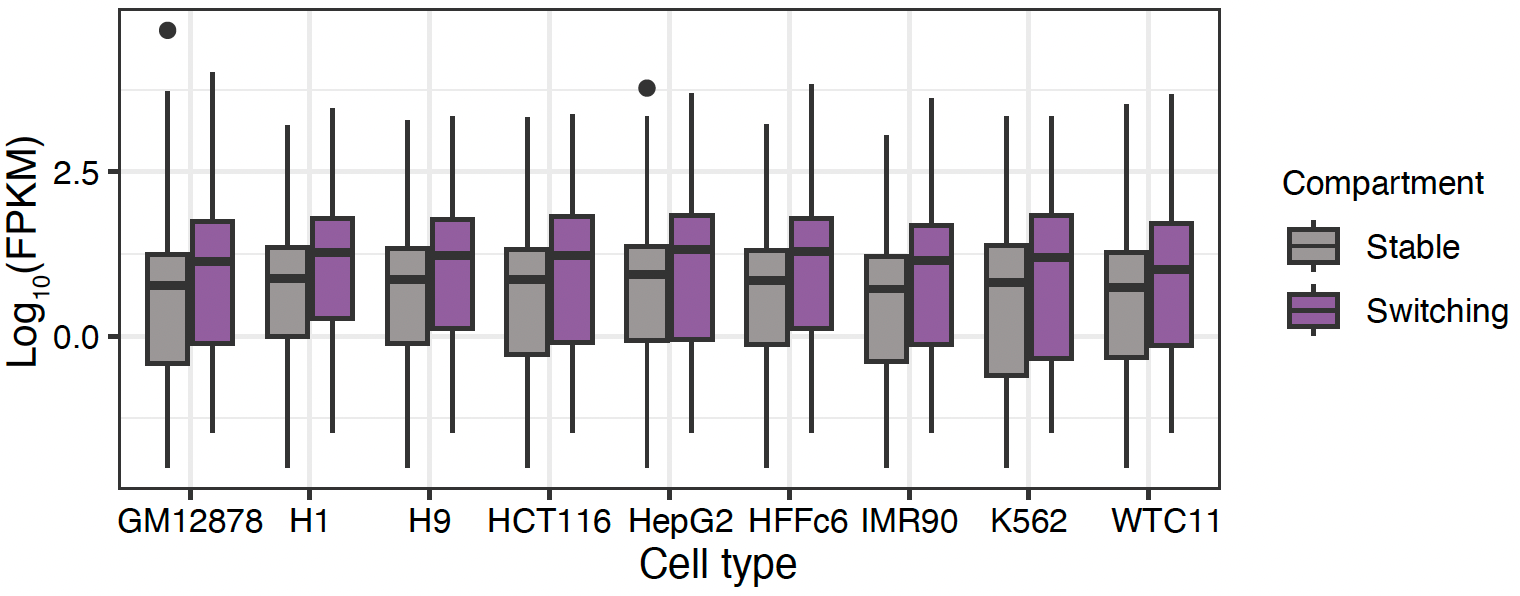


**Figure S3.** Comparison of compartment stability and gene expression levels across cell types.


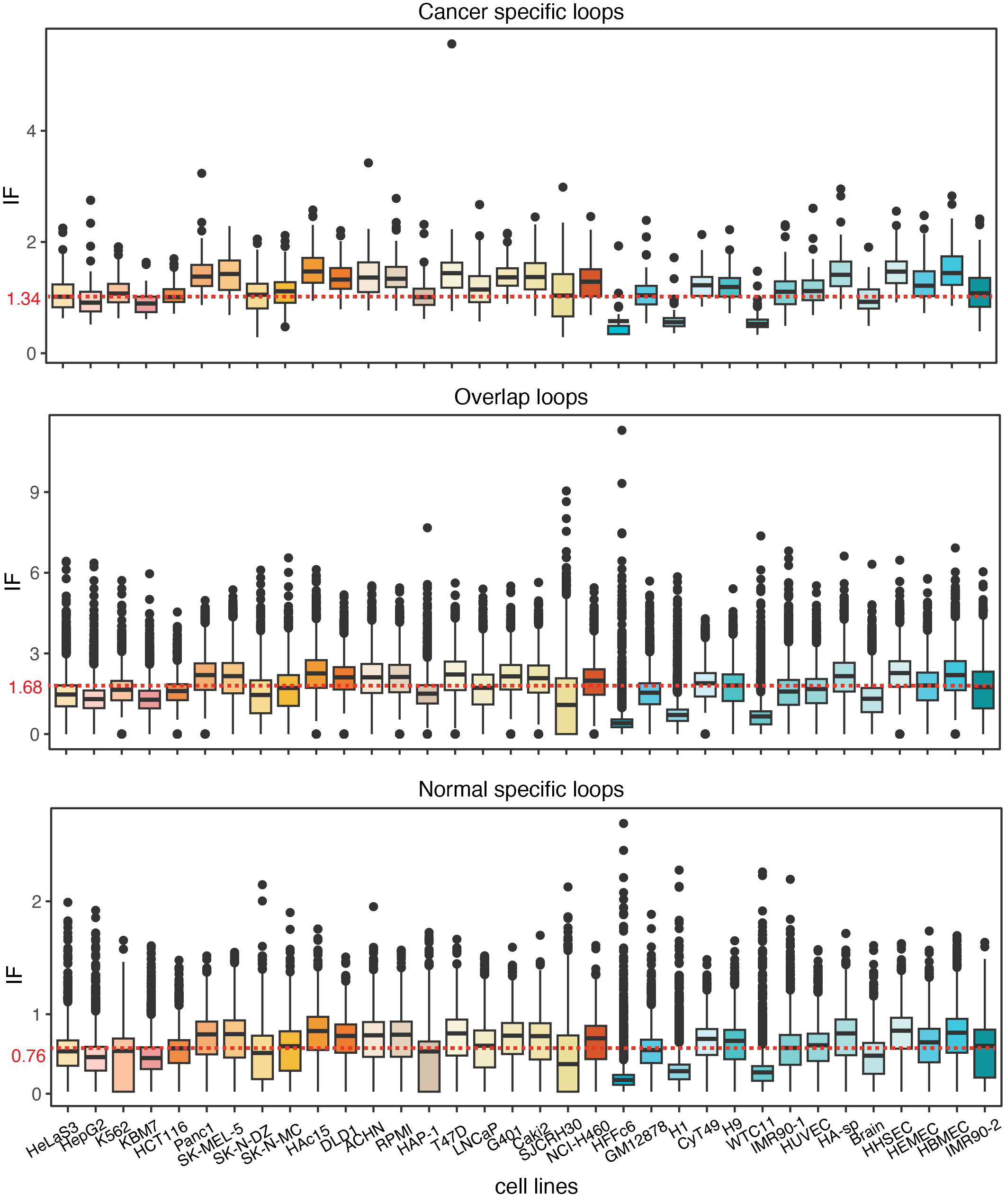


**Figure S4.** Interaction frequency (IF) comparison for cancer-specific loops (CSL), overlap loops (OL), and normal specific loops (NSL) across 34 cell lines. The red dashed lines represent the average IF for each category.

**
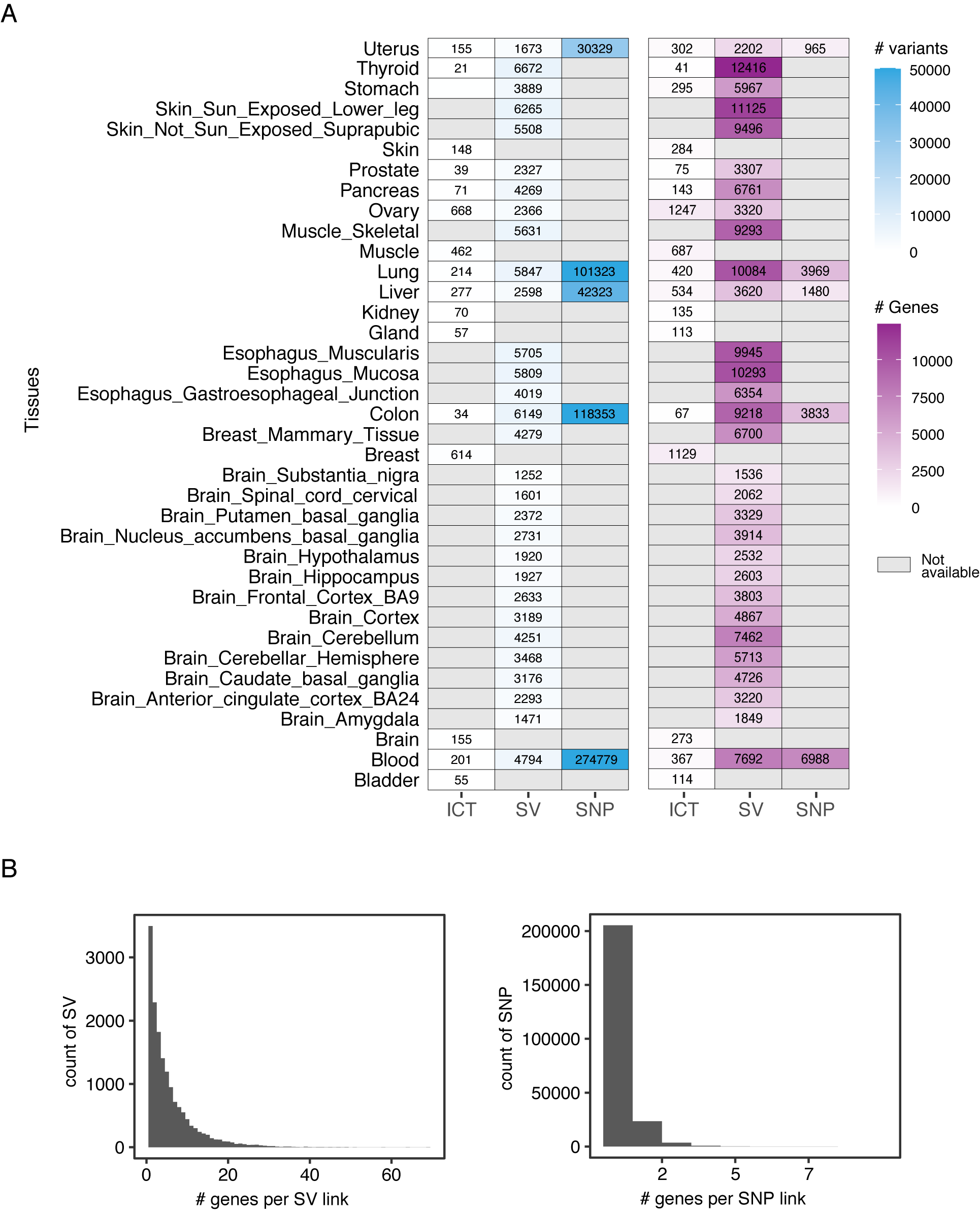
**

**Figure S5. Prediction of ICTs, SVs, SNPs from the PCAWG.** (A) Counts of variants and genes in different tissues. The gray square indicated the result was not available. (B) The number of genes linked to SV/SNP.


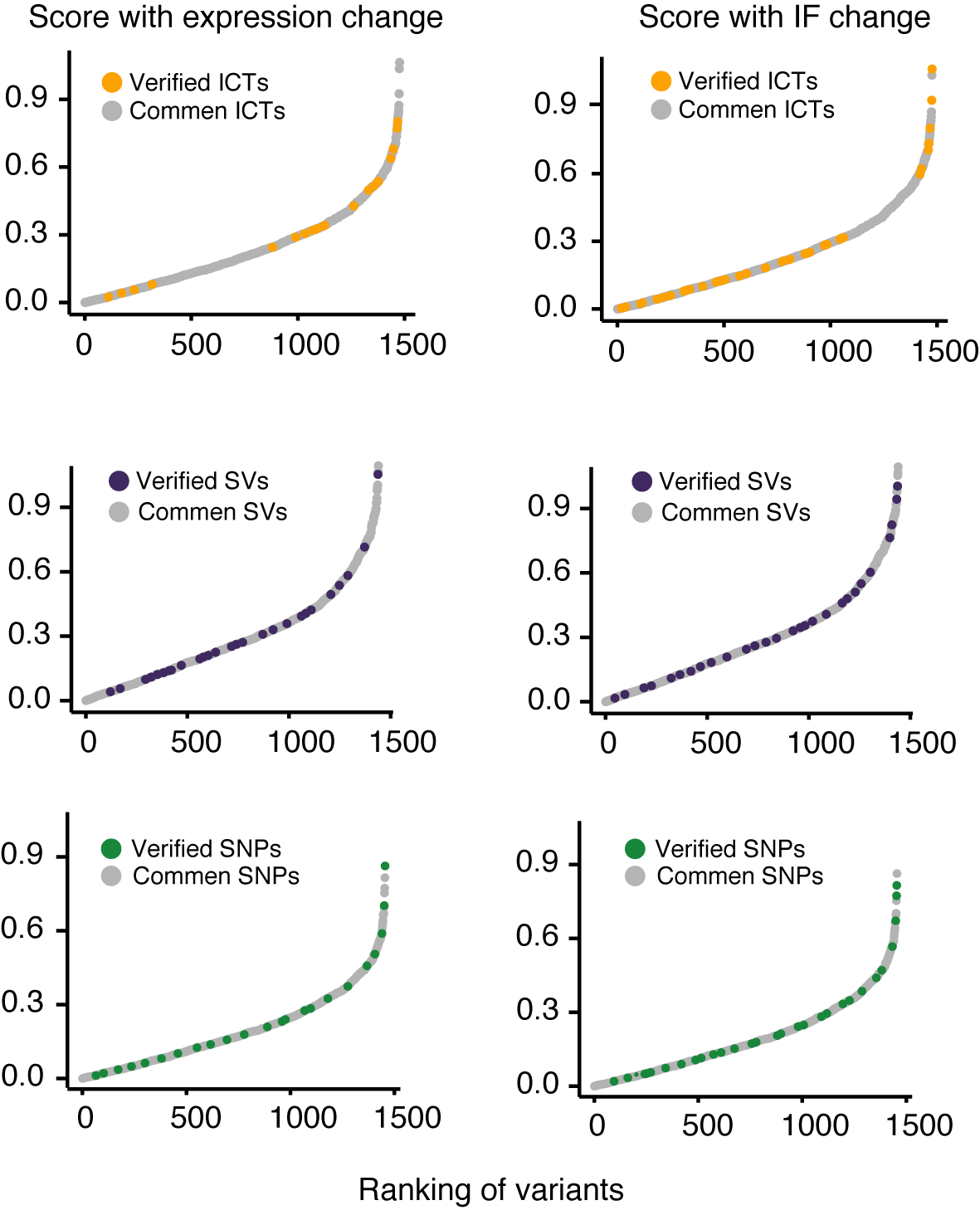


**Figure S6.** Rank variants with expression change or IF change individually.


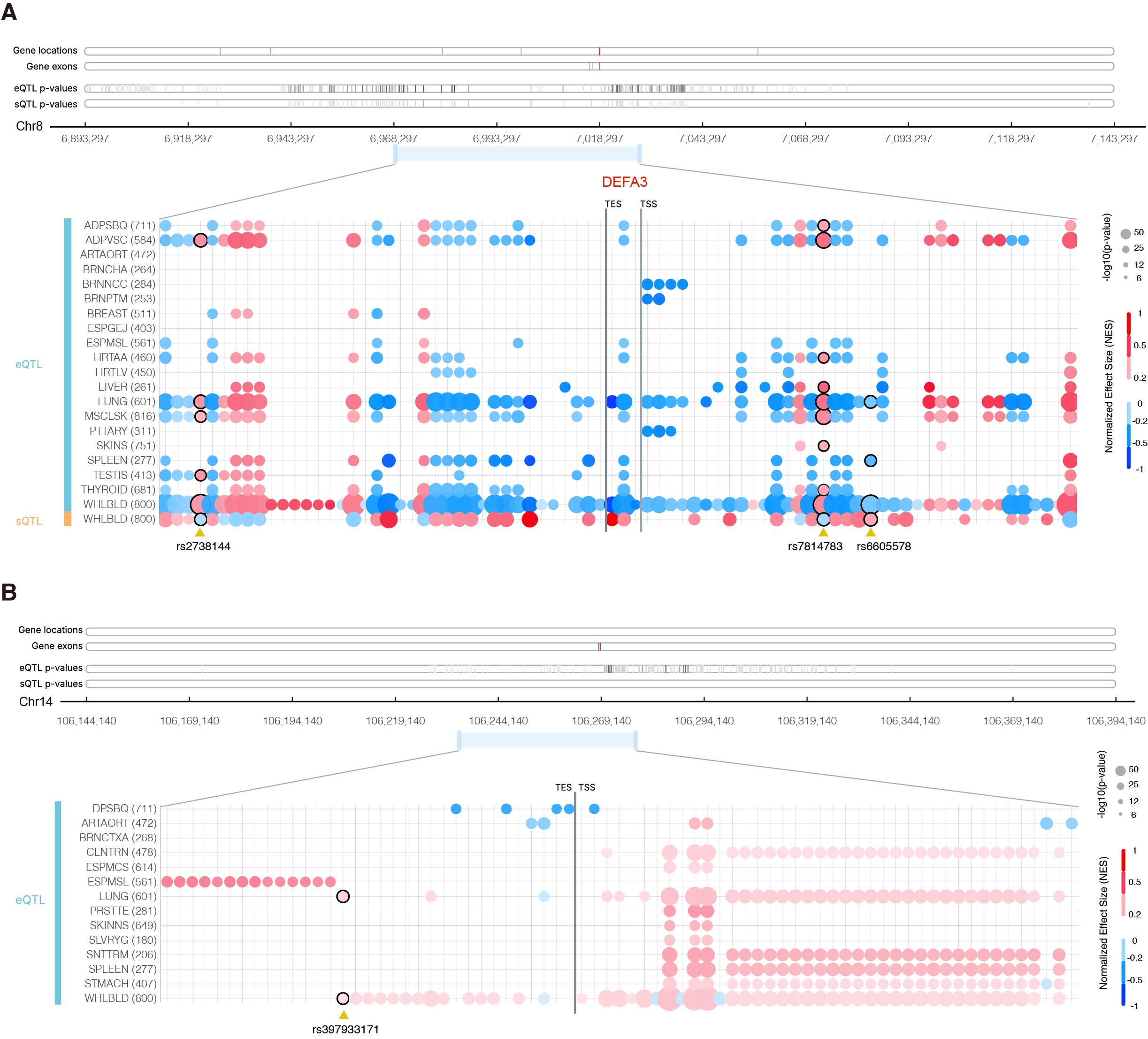


**Figure S7. eQTL and sQTL analysis of SNPs near DEFA3 and IGHV3-23.** (A) eQTL and sQTL analysis of SNP rs2738144 near DEFA3 (Chr8). (B) eQTL analysis of SNP rs397933171 near IGHV3-23 (Chr14).
